# Targeting Mitochondria to Inhibit Aflatoxin Production: Mechanistic Insight

**DOI:** 10.14252/foodsafetyfscj.D-25-00001

**Published:** 2025-09-26

**Authors:** Tomohiro Furukawa, Masayo Kushiro, Hiroyuki Nakagawa, Shohei Sakuda

**Affiliations:** 1 Institute of Food Research, National Agriculture and Food Research Organization (NARO), 2-1-12 Kannondai, Tsukuba, Ibaraki 305-8642, Japan; 2 Research Center for Advanced Analysis, Core Technology Research Headquarters, NARO, 2-1-12 Kannondai, Tsukuba, Ibaraki 305-8642, Japan; 3 Faculty of Science and Engineering, Teikyo University, 1-1 Toyosatodai, Utsunomiya, Tochigi 320-8551, Japan

**Keywords:** aflatoxin, *Aspergillus flavus*, inhibitors, mode of action, mitochondria, respiratory chain complexes

## Abstract

Contamination of agricultural crops by aflatoxin, a potent carcinogenic fungal toxin, is a global issue that poses serious health risks to humans and livestock while inflicting significant economic damage on the agricultural sector. Specific inhibitors of aflatoxin production hold promise not only as effective agents for controlling aflatoxin contamination, but also as valuable tools for uncovering the regulatory mechanisms of secondary metabolism through the elucidation of their modes of action. Unexpectedly, inhibitors whose modes of action we have clarified were found to target mitochondrial components, rather than proteins directly involved in the aflatoxin biosynthetic pathway. In this article, we review inhibitors and inhibitory mixtures that act on mitochondria and explore the relationship between mitochondrial function and aflatoxin production through their modes of action.

## Introduction

Fungi produce a wide range of secondary metabolites, some of which are toxic to human and livestock. These harmful fungal metabolites with a low molecular weight (0.3-0.7 kDa) are collectively referred to as “mycotoxins”^1^^,^^2^^)^. Mycotoxin contamination of food and feed can cause human and animal diseases (mycotoxicoses) and significant economic losses due to food waste. With advances in analytical instrumentation, it was recently estimated that up to 60-80% of food crops produced worldwide contained some kinds of mycotoxins above the limit of detection^3^^)^.

A wide array of fungal species produces mycotoxins with diverse chemical structures, and over 300 mycotoxins have been identified to date. However, six major groups of mycotoxins—aflatoxins, trichothecenes, zearalenone, fumonisins, ochratoxins, and patulin—are frequently detected in agricultural products^4^^)^. Among these, aflatoxins produced by some *Aspergillus* species represented by *A. flavus* and *A. parasiticus* are considered at the most concerning due to their potent carcinogenicity and globally widespread contamination^5^^)^. Aflatoxins are estimated to be responsible for 4.6-28.2% of all newly occurred hepatocellular carcinoma (liver cancer) cases worldwide^6^^)^. In 2004, an outbreak of aflatoxicosis in Kenya resulted in 125 fatalities, due to the high-concentration of aflatoxin-contaminated homegrown maize^7^^)^. Since mycotoxins including aflatoxins are stable during food storage, processing, or cooking at typical temperatures used for food^8^^,^^9^^)^, prevention of contamination is prerequisite. Despite the global prevalence of mycotoxin contamination, effective methods to protect crops from contamination, especially aflatoxin, remain limited.

We have been exploring specific inhibitors of aflatoxin production. Compounds that inhibit aflatoxin production without compromising fungal viability could be valuable for controlling aflatoxin contamination while avoiding the rapid spread of resistant strains, a common concern with fungicides. Such specific inhibitors also serve as molecular tools to investigate the regulatory mechanisms of secondary metabolite production. A deeper understanding of these mechanisms will help identify optimal targets for aflatoxin control. To date, various substances have been screened from microbial metabolites, plant constituents, and synthetic compounds. Detailed reviews of these substances are available in the works of Holmes et al.^10^^)^, Sakuda^11^^)^, Sakuda et al.^12^^)^, Ahmad et al.^13^^)^, and Yoshinari^14^^)^.

We have been attempting to elucidate the mode of action of the aflatoxin production inhibitors identified in our laboratory. Surprisingly, some substances for which we clarified their mechanism of action were found to target mitochondrial components, rather than proteins of the aflatoxin biosynthetic pathway as we had originally assumed. Mitochondria are integral eukaryotic organelles involved in diverse cellular function including energy production, lipid metabolism, calcium homeostasis, signal transduction, and apoptotic activation^15^^)^. However, a direct relationship with aflatoxin production have not been clarified. Therefore, elucidating the modes of action of these inhibitors could uncover novel regulatory mechanisms of aflatoxin production.

**Fig. 1.**
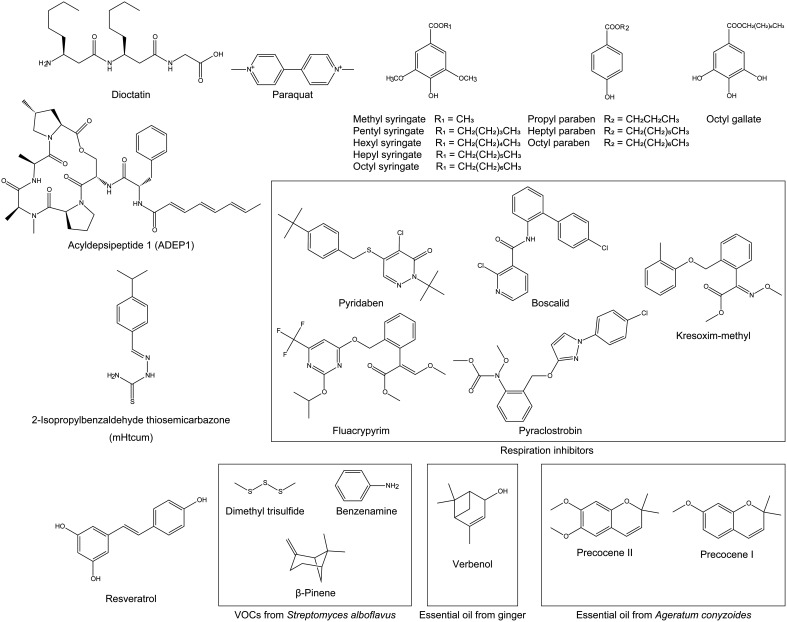
Aflatoxin production inhibitors and mixtures targeting mitochondria For respiration inhibitors, those with low IC_50_ values (<0.1 µM) are shown. For mixtures, the primary components considered to inhibit aflatoxin production are shown.

In this review, we focus on the reported aflatoxin production inhibitors and inhibitory mixtures that target mitochondria, shown in Fig. 1. While the individual inhibitors are highly selective and have little or no effect on mycelial growth, the mixtures are not specific to aflatoxin production and can also inhibit mycelial growth. We then explore the relationship between aflatoxin production and mitochondrial function based on the mechanisms of these inhibitors.

## Aflatoxin Production Inhibitors Targeting Mitochondrial Components

### 1. Dioctatin

Dioctatin is a water-soluble derivative of dioctatin A, a secondary metabolite obtained from *Streptomyces* sp. SA-2581. Dioctatin A was originally discovered as an inhibitor of human dipeptidyl peptidase II (DPP II). Later, Yoshinari et al. reported that it inhibits aflatoxin production without affecting mycelial growth^16^^)^. In PDB liquid medium, dioctatin inhibits AFB_1_ production of *A. flavus* at an IC_50_ value of 23.9 µM^17^^)^. Using dioctatin-chemically immobilized nanobeads, the proteolytic subunit of mitochondrial Clp protease (ClpP) was purified and identified as a specific dioctatin-binding protein. ClpP is localized in the mitochondrial matrix and forms a complex with the chaperone ClpX. Under normal conditions, ClpX unfolds proteins targeted for degradation and delivers them to the proteolytic core at the center of the ClpP tetradecamer complex, where degradation occurs^18^^)^. Consequently, ClpP alone is typically capable of degrading only short peptides. However, in the experiments with recombinant ClpP, dioctatin enables ClpP to degrade substrate proteins without chaperones. Furthermore, it was found that the dioctatin-bound ClpP selectively degrade subunits of mitochondrial respiratory chain complexes I, II, and V, as well as tricarboxylic acid (TCA) cycle enzymes including citrate synthase and malate dehydrogenase. Consistently, when *A. flavus* is cultured with dioctatin, the ATP synthase F1β subunit of complex V is decreased at the protein level. Metabolome analysis revealed that TCA cycle metabolites, such as succinate, malate, and fumarate were reduced in the *A. flavus* cultured with dioctatin. In contrast, the glycolytic intermediate 3-phosphoglycerate was increased.

Dioctatin also influenced gene expression in *A. flavus*, reducing mRNA levels of several aflatoxin biosynthetic cluster genes including *aflD* (*nor-1*), *aflM* (*ver-1*), and *aflH* (*adhA*), while increasing mRNA levels of glycolytic enzyme genes and ethanol fermentation-related enzyme genes, including *adh1*. Additionally, dioctatin treatment decreased histone acetylation levels, which may contribute to these gene expression changes. These findings suggest that dioctatin disrupts the core components of the mitochondrial energy production system, leading to a metabolic shift and suppression of aflatoxin production. It is hypothesized that the minimum energy required for survival is maintained through anaerobic respiration, including ethanol fermentation.

Although the direct application of dioctatin in real agricultural settings is considered impractical due to the expected high costs, a biocontrol approach using *Streptomyces* strain that produce dioctatin A—presumed to share the same mode of action as dioctatin—is promising. This approach involves applying the strain in the field or storage conditions, with the expectation of sustained dioctatin A release to inhibit aflatoxin production. However, several challenges must be addressed before practical implementation, including comprehensive assessment of the safety of dioctatin A and its producing strain, evaluation of potential effects on non-target organisms, and confirmation that sufficient *in situ* production of dioctatin A can be maintained under variable environmental conditions.

### 2. Acyldepsipeptide 1 (ADEP1)

Acyldepsipeptides (ADEPs) are antibiotics found from *Streptococcus hawaiiensis*. Experiments with ADEP-resistant mutants of *Escherichia coli* and *Bacillus subtilis* and affinity purification using ADEP column have identified ClpP as a specific molecular target of ADEPs^19^^)^. ADEPs exert antibacterial activity by abnormally activating ClpP to degrade essential bacterial proteins such as cell division protein FtsZ^20^^)^. Because ADEPs exhibit particularly potent antibacterial activity against Gram-positive bacteria, including the multidrug-resistant *Staphylococcus aureus*, they are expected to become a new class of antibiotics. ADEP1 exhibited a similar effect on the recombinant ClpP of *A. flavus*, causing substrate protein degradation without chaperones^17^^)^. Furthermore, ADEP1 inhibited aflatoxin production at lower concentrations than dioctatin, with an IC_50_ value of 4.9 μM. These results suggested the commonality of structure and function of ClpP among bacteria and fungi and strongly support the predicted mode of action of dioctatin in the inhibition of aflatoxin production.

### 3. Paraquat

Paraquat is a non-selective herbicide. In addition to agricultural fields, it has been utilized in non-agricultural settings such as public spaces, roadsides, and industrial areas. However, due to significant safety and environmental concerns arising from the high toxicity of paraquat, its use has been either banned or strictly regulated in many countries. Paraquat toxicity arises from its role as a redox-cycler, generating superoxide in mitochondria^21^^)^. Within the mitochondrial inner membrane, paraquat undergoes one-electron reduction by mitochondrial respiratory chain complex I to form a paraquat radical, which subsequently reacts with oxygen to produce superoxide. The generated superoxide damages biomolecules such as DNA, proteins, and lipids with high reactivity, leading to their functional loss and extensive mitochondrial damage.

Paraquat inhibited aflatoxin production of *A. flavus* with an IC_50_ value of 54.9 µM^22^^)^. The mycelial growth was not significantly reduced by the addition of 500 µM paraquat, indicating that the mode of action of paraquat is highly selective to aflatoxin production. Paraquat reduced the expression of genes in the aflatoxin biosynthetic gene cluster, specifically the transcriptional regulator gene *aflR* and the enzymatic genes *aflC* (*pksA*), *aflD* (*nor-1*), *aflP* (*omtA*), and *aflQ* (*ordA*). The inhibition of aflatoxin production of paraquat was reverted by co-addition of the antioxidant sodium ascorbate (vitamin C sodium salt) and the Cu/Zn superoxide dismutase, suggesting that the activity of paraquat is due to superoxide generation and subsequent oxidative damage. In fact, observation of mitochondrial superoxide using superoxide-specific mitochondria-localized fluorescent dyes showed that the addition of paraquat increased mitochondrial superoxide in *A. flavus*. These results suggest that paraquat causes accumulation of superoxide and oxidative damage in mitochondria, which leads to decreased aflatoxin biosynthetic cluster gene expression and inhibition of aflatoxin production.

### 4. Alkyl Syringates, Alkyl Parabens, and Alkyl Gallates

Methyl syringate isolated from essential oil of *Betula alba* was found to have weak inhibitory activity against aflatoxin production of *Aspergillus parasiticus* with an IC_50_ value of 0.9 mM^23^^)^. To investigate the structure-activity relationship, a series of alkyl syringates with alkyl chains from methyl to octyl were prepared. Among these, pentyl, hexyl, heptyl, and octyl syringates almost completely inhibited aflatoxin production of *A. flavus* at 0.05 mM^24^^)^. Alkyl paraben and alkyl gallate, analogs of alkyl syringates, also showed aflatoxin production inhibitory activity with propyl to octyl parabens and octyl gallate, respectively.

It has been reported that alkyl gallates with alkyl chains from pentyl to nonyl inhibit mitochondrial complex II activity, with longer alkyl chains exerting stronger effects^25^^)^. Therefore, the complex II inhibitory effect of alkyl syringates was examined. As a result, alkyl syringates with alkyl chains from propyl to octyl inhibited complex II activity, with the longer the alkyl chain, the stronger the effect. These findings suggest that alkyl syringates and their analogs reduce aflatoxin production by inhibiting mitochondrial complex II activity. However, octyl syringate, octyl paraben, and octyl gallate did not reduce the expression of *aflR* or *aflC* (*pksA*), suggesting that these compounds may inhibit aflatoxin production through a pathway that does not involve modulation on gene expression—possibly by affecting the metabolic flow of key molecules.

### 5. Respiration Inhibitors

Screening of the natural products library revealed that siccanin, an inhibitor of mitochondrial complex II, inhibits aflatoxin production of *A. parasiticus*^26^^)^. Therefore, the inhibitory activity of known natural and synthetic inhibitors of respiratory chain complexes I, II, and III against aflatoxin production has been investigated. For natural inhibitors, rotenone (a complex I inhibitor), siccanin and atpenin A5 (complex II inhibitors), and antimycin A (a complex III inhibitor), inhibited aflatoxin production with similar IC_50_ values of around 10 μM. For the synthetic miticides, pyridaben, tolfenpyrad (complex I inhibitors), and fluacrypyrim (a complex III inhibitor) strongly inhibited aflatoxin production with IC_50_ values of less than 0.2 μM. For the synthetic fungicides, boscalid (a complex II inhibitor) showed the strongest activity with an IC_50_ value of less than 0.01 μM. Pyribencarb, kresoxim-methyl, azoxystrobin, and pyraclostrobin (complex III inhibitors) showed inhibitory activity with IC_50_ values of less than 0.5 μM. All of these inhibitors did not inhibit mycelial growth of *A. parasiticus* significantly at the concentrations tested, indicating that inhibition of the respiratory complexes exerts a selective effect on aflatoxin production. No clear correlation has been identified between the respiratory complexes targeted by these inhibitors and their effects on aflatoxin production. Boscalid and tolfenpyrad did not affect the expression of aflatoxin biosynthetic cluster genes^12^^)^.

The IC_50_ value of boscalid for inhibiting aflatoxin production is the lowest among the inhibitors reported to date, suggesting its promising potential for future applications. We are conducting field experiments in peanut farms where aflatoxin contamination of harvested peanuts frequently occurs. In these fields, agrochemicals containing mitochondrial respiratory chain inhibitors, such as boscalid, are applied to peanut leaves before harvest. After harvesting, drying, and storage, aflatoxin levels in treated peanuts are compared with those in untreated controls to evaluate whether even trace amounts of the inhibitors translocate from leaves to kernels and effectively suppress aflatoxin accumulation. Preliminary results suggest that these agrochemicals reduce aflatoxin accumulation in stored peanuts (unpublished results).

### 6. 2-Isopropylbenzaldehyde Thiosemicarbazone

The cuminaldehyde thiosemicarbazone derivatives have been found to have fungistatic and anti-aflatoxigenic activity^27^^)^. In particular, its meta-isopropyl derivative, 2-isopropylbenzaldehyde thiosemicarbazone (mHtcum), has shown strong aflatoxin production inhibitory activity with minimal effect on mycelial growth. The addition of mHtcum led to a reduction in mRNA levels of aflatoxin biosynthetic cluster genes, including *aflO* (*omtB*). Furthermore, it decreased the protein levels of AflO (OmtB), alcohol dehydrogenase (Adh1), and malate dehydrogenase, an enzyme of the TCA cycle.

In subsequent studies, their group identified mitochondrial complex III as the molecular target of mHtcum by biochemical and computational analysis^28^^)^. According to their molecular docking simulations, mHtcum likely binds to complex III’s ubiquinone-reduction (Qi) site which is located on the matrix side of complex III and is the site where ubiquinone binds to be reduced to ubiquinol^29^^)^. Binding of mHtcum in Qi binding pocket obstruct ubiquinone reduction and halt subsequent electron transfer and proton pump function, resulting in loss of the proton gradient between the intermembrane space and matrix and inhibition of ATP production. Antimycin A, a natural complex III inhibitor described above, is known to bind to the Qi site and inhibit ubiquinone reduction^30^^)^, supporting the notion that mHtcum inhibits aflatoxin production by targeting complex III. However, while antimycin A did not affect sclerotia production of *A. flavus*, mHtcum inhibited it. Furthermore, antimycin A did not reduce expression of aflatoxin biosynthetic cluster genes, including *aflR*, *aflD* (*nor-1*), and *aflO* (*omtB*), whereas mHtcum reduced their expression. These differences in the overall regulatory mechanisms suggest that mHtcum targets multiple sites beyond complex III.

### 7. Alternative Oxidase (AOX) Inhibitors

AOX is an integral monotopic membrane protein localized on the matrix side of the inner mitochondrial membrane^31^^)^. AOX mediates the direct coupling of ubiquinol oxidation and O_2_ to H_2_O reduction, thereby making a branch in cytochrome C-based electron transport by mitochondrial complex III. In other words, AOX can bypass the complex III-mediated electron transfer targeted by mHtcum and antimycin A described above. In *Aspergillus nidulans* mutants whose AOX gene was disrupted and overexpressed, the production of sterigmatocystin, the penultimate precursor of aflatoxin B_1_, was largely decreased and increased, respectively^32^^)^. Considering the commonality between aflatoxin production and sterigmatocystin production, AOX function may be also related to aflatoxin production of *A. flavus*. Therefore, the aflatoxin production inhibitory activity of the AOX inhibitors is expected^33^^)^. In fact, resveratrol, which has been reported to inhibit AOX in ciliates, also inhibits aflatoxin production in *A. flavus* by suppressing the expression of aflatoxin biosynthetic cluster genes, including *aflA* (*fas-2*) and *aflB* (*fas-1*)^34^^,^^35^^)^. AOX is absent in mammalian cells and is widely conserved across the plant and fungal kingdoms, making it an attractive target to limit aflatoxin production. However, the low selectivity of the currently available AOX inhibitors, along with its conservation in plant cells, present significant challenges. Therefore, the development and screening of highly selective inhibitors are essential.

## Aflatoxin Production Inhibitory Mixtures Affecting Mitochondria

### 1. Volatile Organic Compounds (VOCs) from *Streptomyces Alboflavus*

VOCs are typically a complex mixture of highly volatile low molecular weight compounds that are quickly biodegraded and often have low environmental persistence. Therefore, they have attracted attention as a control agent against harmful fungi, including *A. flavus*. Yang et al. reported inhibitory activity of mycelial growth, sporulation, conidial germination, and aflatoxin production of *A. flavus* in VOCs emitted by *S. alboflavus* TD-1^36^^)^. *S. alboflavus* TD-1 were cultured in solid medium and VOCs produced were analyzed by GC-MS to identify each compound. Dimethyl trisulfide (DMTS), benzenamine, β-pinene, and anisole were identified as the major constituent of VOCs of *S. alboflavus* TD-1. Exposure of *A. flavus* to the VOCs reduced the fluorescence of rhodamine 123, a fluorescent dye specific for detecting mitochondrial membrane potential. This suggests that the VOCs impairs mitochondrial membrane potential, a critical factor for effective ATP generation and mitochondrial homeostasis^37^^)^. As a likely consequence, the VOCs treatment decreased the expression of aflatoxin biosynthetic cluster genes including *aflR*, *aflS*, *aflD* (*nor-1*), *aflM* (*ver-1*), *aflP* (*omtA*), and *aflQ* (*ordA*).

Among the constituents of the VOCs, dimethyl trisulfide and benzenamine showed potent antifungal activity against *A. flavus*, followed by β-pinene. However, the mode of action of each constituent remains unclear, and it has not been confirmed whether their effects on mitochondria are directly responsible for the inhibition of aflatoxin production. Moreover, it is unlikely that a single component of the VOCs accounts for all the observed effects; rather, additive or synergistic actions are likely involved. Yang et al. suggested that *S. alboflavus* TD-1 may be suitable for use as a biopesticide to control aflatoxin contamination caused by *A. flavus*.

### 2. Essential Oil from Ginger (*Zingiber Officinale* Roscoe)

*Z. officinale* Roscoe is one of the oldest medicinal herbs, and its essential oil (*Zingiber officinale* essential oil, ZOEO) has antifungal activity against a wide range of fungi, including *A. flavus*^38^^)^. ZOEO inhibited mycelial growth of *A. flavus* at MIC of 0.6 µl/ml and its AFB_1_ production at 0.5 µl/ml^39^^)^. Thus, ZOEO inhibits both mycelial growth and aflatoxin production at similar concentrations, indicating that it does not have a specific effect on aflatoxin production. According to GC-MS analysis, the main components of ZOEO were verbenol (52.41%), 7-epi-sesquithujene (6.8%), and γ-terpinene (5.18%). Like the VOCs of *S. alboflavus* TD-1 described above, rhodamine 123 staining showed that mitochondrial membrane potential of *A. flavus* was significantly reduced by ZOEO treatment, suggesting that the normal ATP-synthesis function of mitochondria is impaired by ZOEO.

Singh et al. investigated the effects of ZOEO from multiple angles besides mitochondrial membrane potential: ZOEO treatment decreased the production of ergosterol, a major component of the cell membrane, and induced the leakage of cellular ions such as potassium (K^+^), calcium (Ca^++^), magnesium (Mg^++^), suggesting that the permeability of the plasma membrane was altered. In addition, they performed molecular dynamics simulations and suggested that verbenol, the major component of ZOEO, binds to and stabilizes the gene products of aflatoxin biosynthetic cluster genes including *aflD* (*nor-1*), *aflP* (*omtA*), and *aflK* (*vbs*), thereby inhibiting their function. In conclusion, verbenol-chemotype ZOEO inhibited mycelial growth and aflatoxin B_1_ production of *A. flavus* by multiple pathways, including loss of mitochondrial membrane potential. This highlights the simultaneous targeting of various cellular and molecular mechanisms that collectively suppress aflatoxin production.

### 3. *Ageratum Conyzoides* Essential Oil

*Ageratum conyzoides* L. (Asteraceae) is a medicinal plant with various properties, and its essential oil is known for its antimicrobial activity against fungi^40^^)^. Therefore, the effects of essential oil of *A. conyzoides* on *A. flavus* was investigated and its mode of action was studied using transmission electron microscopy (TEM)^41^^)^. GC/MS analysis was performed to identify 7 components, and the major components were precocene II (46.35%), precocene I (42.78%), cumarine (5.01%), and trans-caryophyllene (3.02%). Essential oil of *A. conyzoides* inhibited mycelial growth and aflatoxin production of *A. flavus*, with aflatoxin production being suppressed at lower concentrations (>0.1 μg/mL in YES medium). TEM observation of essential oil-treated *A. flavus* revealed abnormal structural changes in the plasma membrane and membranous organelles, particularly the mitochondria. Treated cells exhibited disrupted mitochondrial internal structures, including reduction in the ridge polarization of mitochondrial cristae, which may be linked to the suppression of the aflatoxin production.

Since precocene II was a major constituent, it may have been a major factor in the observed mitochondrial abnormal structure. Precocene II was found to have strong inhibitory activity against the production of deoxynivalenol (DON), a trichothecene mycotoxin, in *Fusarium graminearum* without affecting fungal growth^42^^,^^43^^)^. We have reported that the putative molecular target of precocene II in *F. graminearum* is the mitochondrial voltage-dependent anion channel (VDAC), one of the major proteins located on the outer mitochondrial membrane^44^^)^. Precocene II increased superoxide level in mitochondria and oxidative damage in mitochondrial proteins probably due to the blockage of VDAC, resulting in a decrease in the DON production. It is necessary to analyze whether inhibition of aflatoxin production of *A. flavus* by precocene II occurs by a similar mechanism to that of *F. graminearum* on DON production, i.e., by increasing mitochondrial superoxide, and whether this inhibition is selective, not affecting mycelial growth.

## Relationship between Mitochondria and Aflatoxin Production

This review focuses on inhibitors and inhibitory mixtures that target mitochondrial proteins or disrupt mitochondrial function. Many inhibitory mixtures reduced the mycelial growth of aflatoxigenic fungi, likely due to the presence of antifungal agents in mixtures. In contrast, single inhibitors typically exhibit minimal effects on fungal growth while maintaining high selectivity on aflatoxin production. The modes of action of the inhibitors discussed in this review are summarized in **Table 1**. Dioctatin induces degradation of mitochondrial proteins, including subunits of respiratory chain complexes II, III, and V, via aberrant activation of the mitochondrial protease ClpP^17^^)^. ADEP may act in a similar manner to dioctatin. Paraquat functions as a redox-cycler, receiving one-electron from complex I and accumulating superoxide within mitochondria^22^^)^. Alkyl syringates, alkyl parabens, and alkyl gallates inhibit complex II activity^24^^)^. Respiratory inhibitors specifically target respiratory chain complexes I, II, or III^26^^)^. 2-Isopropylbenzaldehyde thiosemicarbazone (mHtcum) inhibits complex III^28^^)^. Alternative oxidase (AOX) inhibitors, such as resveratrol, block mitochondrial alternative oxidase, an oxidoreductase that bypasses complex III in the electron transfer system^33^^)^.

**Table 1.** Aflatoxin production inhibitors targeting mitochondrial components

Inhibitors	IC_50_ (µM)	Sp.^a^	Target	Proposed mode of action	Significantly downregulated genes	Ref.
Dioctatin	23.9	*f*	ClpP	Abnormal degradation of mitochondrial proteins	*aflD*, *aflF*, *aflH*, *aflL*, *aflM*	^17^^)^
Acyldepsipeptide 1 (ADEP1)	4.9	*f*	ClpP	Likely similar to dioctatin	NA	^17^^)^
Paraquat	54.9	*f*	Complex I^b^	Accumulation of mitochondrial superoxide	*aflR*, *aflC*, *aflD*, *aflP*, *aflQ*	^22^^)^
Alkyl syringates and related compounds^c^						
Octyl syringate	NA	*f*	Complex II	Complex II inhibition	-	^24^^)^
Octyl paraben	NA	*f*	Complex II	Complex II inhibition	-	^24^^)^
Octyl gallate	NA	*f*	Complex II	Complex II inhibition	-	^24^^)^
2-Isopropylbenzaldehyde thiosemicarbazone (mHtcum)	NA	*f*	Complex II	Complex II inhibition	*aflR*, *aflD*, *aflO* (*omtB*)	^28^^)^
Respiration inhibitors						
Pyridaben	0.01	*p*	Complex I	Complex I inhibition	NA	^26^^)^
Tolfenpyrad	0.18	*p*	Complex I	Complex I inhibition	-	^12^^,^^26^^)^
Rotenone	13	*p*	Complex I	Complex I inhibition	NA	^26^^)^
Boscalid	<0.01	*p*	Complex II	Complex II inhibition	-	^12^^,^^26^^)^
Atpenin A5	9.7	*p*	Complex II	Complex II inhibition	NA	^26^^)^
Siccanin	13	*p*	Complex II	Complex II inhibition	NA	^26^^)^
Pyraclostrobin	0.06	*p*	Complex III	Complex III inhibition	NA	^26^^)^
Kresoxim-methyl	0.06	*p*	Complex III	Complex III inhibition	NA	^26^^)^
Fluacrypyrim	0.07	*p*	Complex III	Complex III inhibition	NA	^26^^)^
Azoxystrobin	0.4	*p*	Complex III	Complex III inhibition	NA	^26^^)^
Pyribencarb	0.43	*p*	Complex III	Complex III inhibition	NA	^26^^)^
Antimycin A	7.2	*p*	Complex III	Complex III inhibition	-	^26^^,^^28^^)^

NA: Data not available.

-: None of the investigated aflatoxin biosynthetic cluster genes showed significantly reduced expression.

^a^Species tested in the literature. *A. flavus* (*f*) and *A. parasiticus* (*p*).

^b^Potentially involved.

^c^Among these compounds, only octyl- is shown here.

These inhibitors differ in their effects on aflatoxin biosynthetic cluster gene expression: Dioctatin suppresses the expression of *afD* (*nor-1*), *aflH* (*adhA*), *aflM* (*ver-1*), but not significantly for *aflR*^17^^)^. Paraquat suppresses the expression of *aflR*, *aflC* (*pksA*), *aflD* (*nor-1*), *aflP* (*omtA*), *aflQ* (*ordA*)^22^^)^. mHtcum decreases the expression of *aflR*, *aflD* (*nor-1*), *aflO* (*omtB*)^28^^)^. On the other hand, boscalid and tolfenpyrad, respiration inhibitors with strong inhibitory activity against aflatoxin production, did not affect gene expressions of aflatoxin biosynthetic cluster genes^12^^)^. Likewise, antimycin A did not decrease the expression of *aflR*, *aflD* (*nor-1*), *aflO* (*omtB*), in contrast to mHtcum^28^^)^. Octyl syringate, octyl paraben, and octyl gallate also did not reduce the expression of *aflR*, *aflC* (*pksA*). The factors responsible for the differences in gene expression effects are unclear. Variations in culture conditions across these studies may contribute, and some inhibitors, such as dioctatin, paraquat, and mHtcum, are considered to have additional sites of action beyond the respiratory chain. These alternative action points may explain the observed effects on gene expression.

**Fig. 2.**
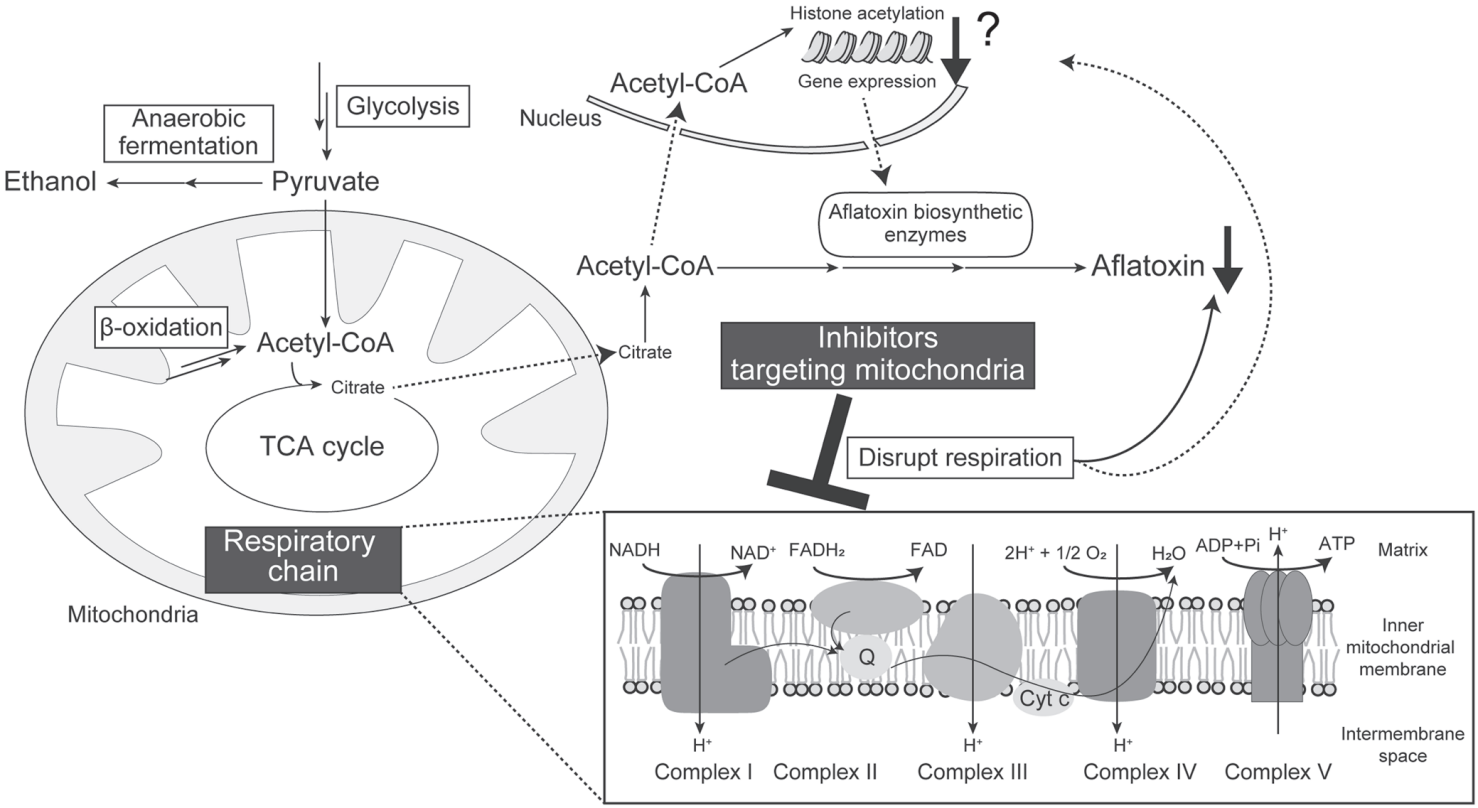
Modes of action of mitochondria-targeting inhibitors These inhibitors commonly induce dysfunction in the mitochondrial respiratory chain, leading to reduced aflatoxin production. While some inhibitors downregulate the expression of aflatoxin biosynthetic cluster genes, others show no significant effect on gene expression. The effects of these inhibitors may be mediated by disruptions in the balance of ATP and acetyl-CoA—the starting point of the TCA cycle and a critical precursor for aflatoxin biosynthesis.

Considering the modes of action of the single inhibitors, inducing a malfunction in the mitochondrial respiratory chain appears to be a common feature of these inhibitors (Fig. 2). How does inhibition of the mitochondrial respiratory chain lead to inhibition of aflatoxin production? The primary functions of the respiratory chain complexes are to facilitate electron transfer and form a proton gradient across the mitochondrial inner membrane, which drives ATP production via oxidative phosphorylation. However, the effects of respiratory chain inhibition are not confined to a reduction in ATP production; they encompass a variety of downstream consequences^45^^)^. Inhibition of complex I (NADH/ubiquinone oxidoreductase) leads to an increased NADH/NAD^+^ ratio due to impaired oxidation of NADH. The elevated NADH/NAD^+^ ratio inhibits dehydrogenase enzymes in the TCA cycle, thereby disrupting the smooth progression of TCA cycle metabolism. Similarly, inhibition of complex II reduces the oxidation of FADH_2_, impairing FADH_2_/FAD balance. The levels of NADPH, which is required in multiple steps of aflatoxin biosynthesis^46^^)^, may also be affected by mitochondrial respiratory dysfunction. As suggested by the mode of action of dioctatin, malfunction of the respiratory chain is thought to enhance glycolytic flux. This metabolic shift may redirect glucose-6-phosphate toward glycolysis rather than the pentose phosphate pathway, potentially reducing NADPH generation^47^^)^. Among the effects induced by respiratory chain malfunction, fluctuations in acetyl-CoA balance may play a key role in the inhibition of aflatoxin production. Acetyl-CoA, the entry substrate for the TCA cycle, serves not only as a building block for aflatoxin biosynthesis but also as an essential donor for histone acetylation—a process required for the initiation of the expression of aflatoxin biosynthetic cluster genes^48^^)^. Within mitochondria, acetyl-CoA is produced via β-oxidation of fatty acids and from pyruvate (the end product of glycolysis) through the action of mitochondrial pyruvate dehydrogenase. Inhibition of the respiratory chain may shift metabolism from mitochondrial respiration to anaerobic fermentation, leading to reduced mitochondrial acetyl-CoA production. This, in turn, reduces citrate export to the cytosol, thereby limiting cytosolic acetyl-CoA availability^49^^)^, reducing histone acetylation, and ultimately suppressing aflatoxin biosynthesis (Fig. 2). These metabolic shifts appear to allow fungal proliferation to be maintained, as if chemically converting aflatoxin-producing strains into non-producing ones.
